# Factors Influencing Stem Subsidence in 135-Degree Inlay Reverse Shoulder Arthroplasty

**DOI:** 10.3390/jcm14207359

**Published:** 2025-10-17

**Authors:** Suguru Mori, Katsumasa Nakazawa, Tomoya Manaka, Yoichi Ito, Yoshihiro Hirakawa, Naoya Kubota, Hidetomi Terai

**Affiliations:** 1 Department of Orthopedic Surgery, Kishima Hon-in Hospital, 3-3-33 Eiwa, Yao-shi 581-0853, Osaka, Japan; suguru.m.93@gmail.com; 2 Department of Orthopedic Surgery, Osaka Metropolitan University Graduate School of Medicine, 1-4-3 Asahimachi, Abeno-ku, Osaka 545-8585, Osaka, Japan; manakatomoya@yahoo.co.jp (T.M.); yoshimpreza@gmail.com (Y.H.); naoyanaoya03134649@gmail.com (N.K.); terai@omu.ac.jp (H.T.); 3 Osaka Shoulder Center, Ito Clinic1-10-12 Ueda, Matsubara-shi 580-0016, Osaka, Japan; yito@omu.ac.jp

**Keywords:** reverse shoulder arthroplasty, stem subsidence, inlay-type humeral stem, filling ratio, stem alignment

## Abstract

**Background/Objectives:** Reverse shoulder arthroplasty (RSA) using a 135-degree inlay-type humeral stem has recently gained popularity due to its bone-preserving design. However, stem subsidence (hereafter, subsidence) and its contributing factors are poorly understood. We aimed to investigate the incidence of subsidence and its associated factors in patients undergoing RSA using a 135-degree inlay-type stem. **Methods:** A total of 44 shoulders treated with uncemented Tornier Perform^®^ Reversed Stems were retrospectively analyzed. Radiographic evaluations included stem alignment and canal filling ratio at three levels. The outcome, subsidence, was defined as >5 mm inferior migration of the stem. **Results:** Subsidence was observed in 6 shoulders (13.6%), which showed significantly greater stem alignment and lower proximal filling ratio. Logistic regression analysis identified proximal filling ratio <80% as an independent risk factor (odds ratio: 70.0, 95% confidence interval: 3.6–1342.6). **Conclusions:** Although the findings remain exploratory due to the small sample size and short follow-up period, they suggest that inadequate proximal fit may contribute to subsidence in 135-degree inlay RSA. Ensuring proper stem sizing and alignment during implantation may be essential to improving initial stability and clinical outcomes. Larger, long-term studies are required to generalize these conclusions.

## 1. Introduction

Reverse shoulder arthroplasty (RSA) has become an established surgical intervention for irreparable rotator cuff tears and cuff tear arthropathy [1]. In the past decades, the humeral stem designs have been refined continuously. In recent years, uncemented short stem prostheses (USSPs) have gained popularity due to their bone-preserving properties [2,3]. Among these, the inlay-type humeral implants with a 135-degree neck-shaft angle have been newly developed and are increasingly being adopted in clinical practice. The Tornier Perform^®^ Reversed Stem (Stryker, Memphis, TN, USA) is an example of such implants. This stem, designed for metaphyseal fixation, features a medial collar for load transfer to the native bone and a tapered distal stem to reduce the canal filling ratio. Additionally, the proximal portion is coated with porous titanium, and rotational stability is enhanced by the lateral flat surfaces and a medial fin.

Although a registry-based study by Zhou et al. demonstrated that both inlay and onlay humeral stem designs provide comparable medium-term clinical outcomes in RSA [4], stem design may influence the development of clinical outcomes, including the risk of stem subsidence (hereafter, subsidence), as suggested in recent comparative studies [5]. In RSA, when using the 135-degree inlay-type humeral implant (135-degree inlay RSA), subsidence has been sporadically observed. Tross et al., who employed a USSP with a tray-based design, reported the occurrence of subsidence in RSA utilizing USSPs [6]. In contrast, the 135-degree inlay RSA examined in the present study uses a stem without a tray structure, which may compromise mechanical stability and potentially lead to a higher incidence of subsidence.

Furthermore, previous studies also suggested that factors such as stem canal filling ratio, alignment, and bone quality may influence stem stability and subsidence occurrence [7,8,9]. Other previous studies also indicated that peri-stem bone remodeling and bone mineral density changes may be associated with stem positioning and fixation [10,11]. However, investigations focused on 135-degree inlay RSA specifically remain limited.

Thus, we proposed the following two hypotheses: first, that 135-degree inlay RSA without a tray structure may be associated with a higher incidence of subsidence; and second, that factors such as stem alignment, canal filling ratio, and the presence of osteoporosis may affect the risk of subsidence. To test these hypotheses, we aimed to evaluate the incidence of subsidence in 135-degree inlay RSA and identify associated factors, with particular emphasis on the radiographic parameters.

## 2. Materials and Methods

### 2.1. Study Design and Patient Selection

In this study, we retrospectively identified patients who underwent RSA using the Tornier Perform^®^ Reversed Stem at a single institution between August 2023 and April 2024 for cuff tear arthropathy or irreparable rotator cuff tears. Patients with a history of revision RSA, proximal humeral fracture sequelae, shoulder dislocation, infection, or acute proximal humeral fractures, were excluded. Only patients with a minimum of 1-year postoperative follow-up were included. Additionally, patients for whom cemented stem was used were excluded from the final analysis. All patients were treated with the short-stem version of this implant.

### 2.2. Surgical Procedure and Rehabilitation

All procedures were performed using the standard deltopectoral approach by three surgeons (Y.H., T.M. and Y.I.). The subscapularis tendon was completely repaired, whenever possible. In addition, bony increased-offset RSA incorporating bone grafting was performed in all patients. All patients underwent the same postoperative protocol with a sling for 2 weeks. Assisted range of motion (ROM) exercise was initiated 2 days postoperatively, and free ROM exercises were initiated after weaning off the sling. The strengthening program began 3 months postoperatively.

### 2.3. Radiographical Measurements

**Figure 1:**
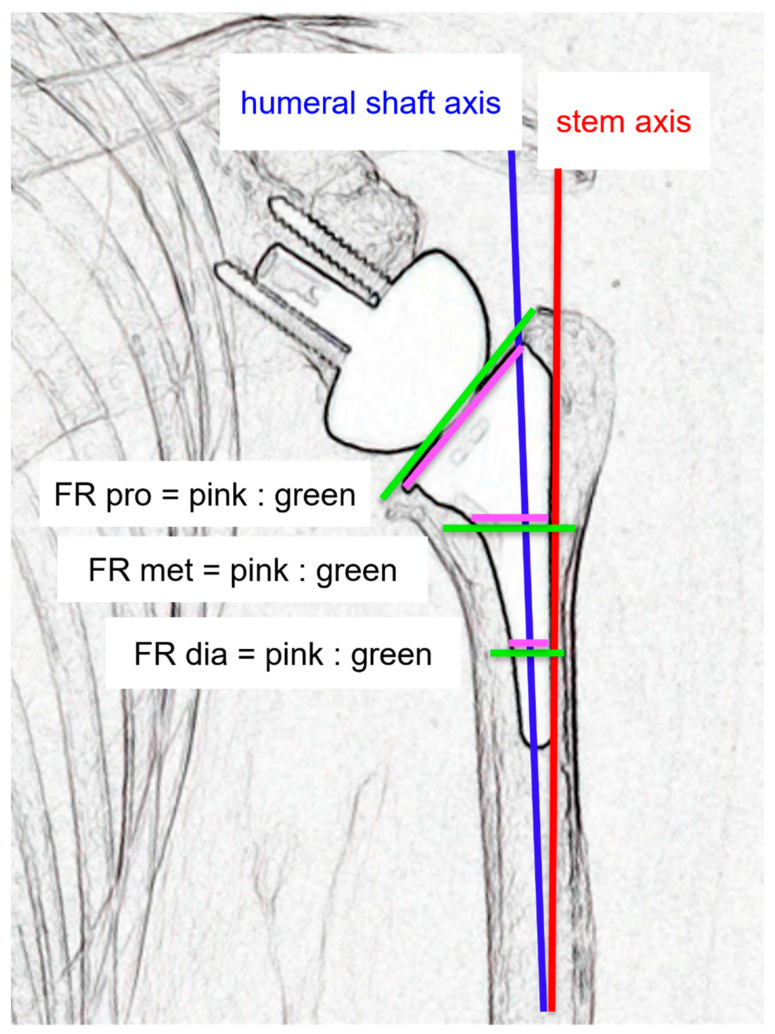
Radiographic measurements. Stem alignment was measured as the angle between the humeral axis (blue line) and the stem axis (red line). Canal filling ratio was calculated at three levels: (1) proximally (filling ratio pro, at the osteotomy site); (2) at the metaphysis (filling ratio met, at a line perpendicular to the humeral axis from the stem’s medial inflection point); and (3) at the diaphysis (filling ratio dia, at a line perpendicular to the humeral axis at the distal third of the stem). At each level, the quotient between the pink and the green line is the filling ratio.

Stem alignment and canal filling ratio were evaluated using anteroposterior humeral radiographs taken immediately postoperatively and at the final follow-up, as described by Raiss et al. [12]. Stem alignment was measured as the angle between the humeral axis and the outer edge of the stem. Filling ratio, calculated at three levels: (1) proximally (filling ratio pro, at the osteotomy site); (2) at the metaphysis (filling ratio met, at a line perpendicular to the humeral axis from the stem’s medial inflection point); and (3) at the diaphysis (filling ratio dia, at a line perpendicular to the humeral axis at the distal third of the stem). The filling ratio for each level was determined by dividing the stem diameter by the intramedullary canal diameter, and reported as percentages (Figure 1).

Subsidence was determined by comparing the distance between the most cephalic aspect of the greater tuberosity with the distal border of the stem, according to Bogle et al., and empirically defined as an inferior migration of the shaft >5 mm [6,13]. To assess subsidence, radiographs taken immediately after surgery and at the final follow-up were compared, and statistical analyses were performed to examine its association with stem alignment and filling ratio.

### 2.4. Statistical Analysis

Continuous variables are expressed as means ± standard deviations, and categorical variables as numbers and percentages. For comparisons between subsidence and non-subsidence groups, the Wilcoxon rank-sum test was applied for continuous variables, and Fisher’s exact test for categorical variables. To identify potential predictors of stem subsidence, univariate logistic regression analysis was performed. Odds ratios (ORs) and 95% confidence intervals (CIs) were calculated. A *p*-value of <0.05 was considered statistically significant. Statistical analyses were performed using SPSS Statistics for Windows, version 25.0 (IBM Corp., Armonk, NY, USA).

### 2.5. Ethics Declarations and Consent to Participate

Approval by the institutional review board was obtained at Osaka Metropolitan University (approval no. 2021-277; approval date: 28 March 2022).

## 3. Results

**Figure 2:**
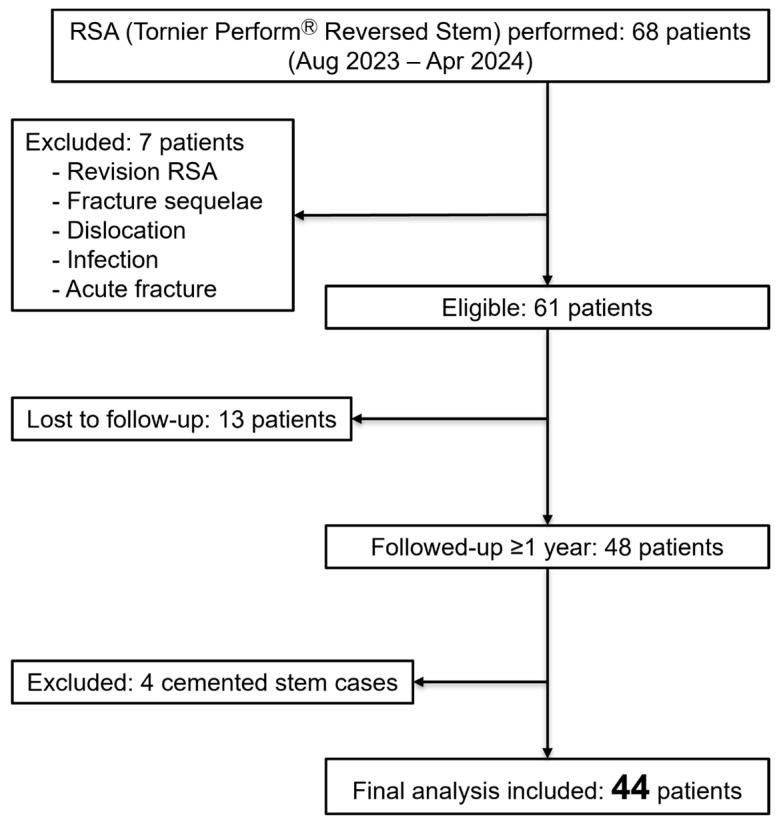
Flow chart of the study cohort. RSA, reverse shoulder arthroplasty.

Of 68 patients that underwent RSA using the Tornier Perform^®^ Reversed Stem, after excluding 20 owing to various conditions and loss-to-follow-up, 48 patients with a minimum postoperative follow-up of 1 year were included. Of these, the four patients who received cemented stems were excluded, leaving 44 patients in the analysis (Figure 2). The 44 patients’ average age was 76.8 ± 6.0 years (range: 64–88), with 25 (56.8%) males. RSA was performed on the right and left sides in 24 and 20 patients, respectively. The average height, weight, and body mass index were 152.2 ± 24.5 cm (range: 138.2–181.0); 57.9 ± 13.3 kg (range: 38.0–82.0); and 23.9 ± 3.4 kg/m^2^ (range: 16.0–30.2), respectively. Among the study participants, eight, six, and ten patients had diabetes mellitus and hypertension or heart disease, and treated for osteoporosis, respectively. Of the 10 patients, two, one, two, two, two, and one received eldecalcitol, bazedoxifene acetate, minodronic acid hydrate, teriparatide, romosozumab, and denosumab, respectively, all administered preoperatively, without discontinuation. The mean follow-up period was 16.0 ± 2.8 (range: 12–20) months. The patient demographic data are presented in Table 1. The subscapularis tendon was and was not repaired in 41 and three patients, respectively.

Among the 44 shoulders, subsidence occurred in six (13.6%) patients, who were all males (*p* = 0.022). The overall mean stem alignment was 3.6 ± 3.7°, and mean filling ratios at proximal, metaphyseal, and diaphyseal levels were 81.0 ± 6.7%, 45.6 ± 6.1%, and 40.6 ± 6.9%, respectively (Table 2). Comparison between the subsidence and non-subsidence groups revealed no significant differences in age, height, weight, body mass index (BMI), or prevalence of osteoporosis and diabetes. However, stem alignment was significantly greater in the subsidence group (6.4 ± 2.8° vs. 3.1 ± 3.7°, *p* = 0.033) and filling ratio pro was significantly lower (78.0 ± 2.2% vs. 81.4 ± 7.1%, *p* = 0.040) (Table 3).

Receiver operating characteristic (ROC) curve analysis revealed cutoff values of 5.5° for stem alignment and 80% for filling ratio pro. Stratified analysis based on these thresholds showed a significantly higher incidence of subsidence in the shoulders with stem alignment ≥5.5° (Table 4, *p* < 0.01), or with filling ratio pro <80% (Table 5, *p* < 0.01).

Logistic regression analysis revealed filling ratio pro as a significant independent predictor of subsidence (odds ratio: 70.0, 95% confidence interval: 3.6–1342.6, *p* < 0.01). In contrast, stem alignment was not (odds ratio: 0.031, 95% CI: 0.99–1.20, *p* = 0.99) (Table 6).

## 4. Discussion

In this study, we investigated the incidence and associated factors of stem subsidence in 44 shoulders treated with 135-degree inlay RSA. Subsidence was observed in six shoulders (13.6%), and a lower filling ratio pro was significantly associated with its occurrence. Logistic regression analysis further identified filling ratio pro as an independent risk factor for subsidence.

These findings are consistent with previous reports by Tross et al. and Raiss et al. [6,12], emphasizing that the fit between the stem and diaphyseal canal is critical for initial implant stability. In particular, with the use of uncemented short stems in RSA, achieving proximal stability is essential, as insufficient canal filling may result in increased micromotion and subsequent stem migration. This concept is further supported by recent biomechanical and clinical studies. Ritter et al. demonstrated through three-dimensional volumetric analysis that lower canal filling ratios significantly increased implant tilt and subsidence under cyclic loading, particularly in short-stem designs, whereas higher filling ratios minimized medial calcar deformation and improved initial fixation [7]. Clinically, Dukan et al. reported that patients undergoing uncemented short-stem RSA achieved satisfactory short-term outcomes with average metaphyseal and diaphyseal filling ratios of approximately 50–60%, but emphasized that achieving adequate metaphyseal engagement is important for stability over time [14].

Regarding stem alignment, although univariate analysis suggested an association with subsidence, it was no longer significant in regression analysis. This implies that the deviation of alignment alone may be an indecisive factor for subsidence, and that structural factors such as proximal support may have a more substantial impact. This concept is further supported by biomechanical testing and clinical evidence. In a cadaveric evaluation using a bespoke short humeral stem, Cho et al. showed that varus positioning increased instability, whereas valgus did not differ significantly from neutral, indicating that alignment can modulate stability but is context-dependent [8]. Complementing this, a clinical series of curved short stems by Lee et al. found that varus/valgus stem alignment did not significantly affect clinical outcomes, supporting our observation that alignment alone may not independently determine migration risk [15]. Furthermore, the study exclusively utilized a 135° inlay-type humeral stem, and in all patients, a bony increased-offset technique incorporating bone grafting was applied. Both factors inherently contribute to lateralization—a biomechanical adjustment known to enhance soft tissue tension and deltoid lever arm. However, the lateralization also increases joint reaction forces, which can be particularly problematic in the presence of poor bone quality or inadequate initial fixation. These elevated forces potentially heighten the risk of subsidence. Prior biomechanical reviews have emphasized that humeral-side lateralization, such as bony increased-offset RSA, can alter muscle loading and implant torque characteristics [16]. Furthermore, radiographic analyses highlight that lateralized RSA designs—whether glenoid or humeral—can modify load transfer and fixation patterns, informing implant stability considerations [17].

Clinically, our findings suggest that the selection of an appropriate stem size to achieve at least 80% proximal canal filling intraoperatively is important for preventing subsidence. Although malalignment should not be overlooked, it may not independently determine subsidence risk and should be considered alongside canal filling during stem implantation. However, Han et al. identified malalignment, longer stems, and higher diaphyseal filling ratios as independent risk factors for proximal stress shielding, with malalignment showing the highest odds ratio [18]. This finding appears to contrast with our results, as it emphasizes malalignment and highlights potential adverse effects of higher filling ratios. Similarly, de Joode et al. reported that higher diaphyseal filling ratios in RSA were associated with increased bone resorption and a higher risk of intraoperative fractures [19], while Vasiliadis et al. concluded in a systematic review that poor coronal alignment and higher filling ratios could increase stress shielding, potentially compromising long-term implant stability [10]. These differences may, in part, be explained by variations in stem design. Previous studies often involved long or curved stems, whereas our series exclusively utilized a tapered distal stem. This design may allow for secure metaphyseal fixation while reducing distal stiffness, thereby mitigating the negative effects of higher proximal filling observed in other stem geometries. We believe this distinction underlies the novelty of our findings and supports the conclusion that, for tapered distal stems, achieving sufficient proximal engagement remains a priority to reduce subsidence risk. On the other hand, as reported, excessive filling ratios increase the risk of stress shielding and proximal bone resorption [12,20]. Therefore, while it is important to achieve adequate proximal filling, a higher filling ratio is not necessarily better; achieving a balanced fit is desirable.

In our study, we encountered patients with subsidence who exhibited a shortened distance between the greater tuberosity and acromion, and showed positive signs of subacromial impingement. While we did not evaluate the clinical scores, it remains possible that subsidence could contribute to impingement and altered deltoid tension, potentially leading to functional decline in the mid- to long-term. Thus, future studies should investigate the relationship between subsidence, subacromial space, clinical outcomes, and muscle strength, in a multifaceted manner. Moreover, although subsidence may potentially influence the clinical outcomes through impingement or altered deltoid tension, we did not assess the clinical outcome data in this study, and therefore this discussion remains hypothetical.

Interestingly, all six patients with subsidence in this study were males, suggesting that sex may be a contributing factor. Generally, males have greater skeletal muscle mass and tend to be more physically active postoperatively, potentially increasing mechanical stress on the implant. This hypothesis is indirectly supported by registry-based studies. Hung et al. reported that male sex was associated with higher rates of early revision and complication following shoulder arthroplasty [21]. Furthermore, Lehtimäki et al., using the Nordic Arthroplasty Register Association (NARA) database, reported a higher risk of revision in male patients after RSA [22]. Conversely, Hochreiter et al. observed that female patients tended to achieve slightly lower functional outcomes following reverse RSA [23], suggesting that the effect of sex is not straightforward. Chang et al., in an analysis of 227 stemless TSAs, reported no significant differences in clinical outcomes or implant migration between sexes [24]. Their study highlighted that female patients are more susceptible to bone-related complications such as loosening and periprosthetic fractures, likely due to osteoporosis. In contrast, male patients tended to be more prone to mechanical complications and revision surgeries, underscoring the complex nature of sex-related differences. In addition, a systematic review and meta-analysis of the shoulder anatomical and functional sex differences demonstrated that, compared with men, women tend to have smaller glenoid diameters, weaker peri-shoulder muscle strength, and greater ranges of motion, which may influence implant stability and load transfer [25]. Taken together, these findings suggest that in men, increased mechanical loads associated with muscle strength and activity may heighten the risk of subsidence. However, in women, bone-related complications associated with poor bone quality may predominate. The number of subsidence cases in our cohort was small, and these interpretations should be made with caution. Larger studies are warranted for validation.

This study has some limitations. First, it was a retrospective study with a relatively small sample size, particularly in the subsidence group (n = 6), which may have introduced statistical bias. The very wide confidence intervals of the odds ratios indicate uncertain estimates, and the logistic regression results should be regarded as exploratory. Second, radiographic evaluations were based solely on plain radiographs, which may not accurately reflect three-dimensional positioning or bone quality. In addition, the mean follow-up period of 16 months was relatively short, and long-term risks of subsidence or revision could not be assessed. Third, there is no universally accepted definition or clinical threshold for subsidence, and the functional implications of a minor migration remain unclear. Moreover, because the clinical outcome data were not collected, we could not fully examine the clinical significance of the radiographic findings. Although the >5 mm threshold for subsidence was based on the previous literature [6,13], the reported incidence may vary according to definitions. Measurement reproducibility was also not quantitatively assessed in this study. Future research should focus on larger patient populations and long-term outcomes. In addition, three-dimensional assessments using CT imaging may help to further elucidate the mechanisms underlying subsidence.

## 5. Conclusions

This study demonstrated that stem subsidence occurred in approximately 13.6% of shoulders undergoing 135-degree inlay RSA. Our results suggest that insufficient proximal filling may be associated with an increased risk of subsidence, although alignment should still be considered intraoperatively. However, given the small sample size and short follow-up, this study’s cutoff values and predictive factors should be regarded as exploratory findings. Therefore, while surgeons should pay attention to achieving adequate proximal filling and proper alignment during preoperative planning and implantation, larger and longer-term studies are needed to generalize these conclusions.

## Figures and Tables

**Table 1:** The study cohort’s characteristics.

Characteristics	Values
Shoulders, *n*	44
Age (years)	76.8 ± 6.0
Sex, female/male, *n* (%)	19 (43.2)/25 (56.8)
Operated side, right/left, *n* (%)	24 (54.5)/20 (45.5)
Height (cm)	152.2 ± 24.5
Weight (kg)	57.9 ± 13.3
BMI (kg/m^2^)	23.9 ± 3.4
Cuff tear arthropathy, *n* (%)	18 (40.9)
Massive rotator cuff tears, *n* (%)	26 (59.1)
Uncemented RSA, *n* (%)	44 (100)
Bio-RSA, *n* (%)	44 (100)
Diabetes mellitus, *n* (%)	8 (18.1)
Hypertension or heart disease, *n* (%)	6 (13.6)
Osteoporosis, *n* (%)	10 (22.7)
Follow-up (months)	16.0 ± 2.8

The following characteristics are reported as means and standard deviation: (BMI, body mass index; Bio-RSA, bony increased offset reversed shoulder arthroplasty; RSA, reverse shoulder arthroplasty).

**Table 2:** Radiological outcomes including stem subsidence, alignment, and canal filling ratios.

Radiological Outcome	Values
Subsidence, *n* (%)	6 (13.6)
Stem alignment (degrees)	3.6 ± 3.7
FR pro (%)	81.0 ± 6.7
FR met (%)	45.6 ± 6.1
FR dia (%)	40.6 ± 6.9

FR, filling ratio; pro, proximal; met, metaphyseal; dia, diaphyseal. Radiological parameters are presented as mean ± standard deviation.

**Table 3:** Comparison of demographic and radiological characteristics between patients with and without stem subsidence.

	Subsidence		
	No	Yes	*p* Value
Shoulders, *n*	38	6	
Age (years)	77.1 ± 6.0	75.6 ± 7.3	0.619
Sex, female/male, *n* (%)	17 (44.7)/21 (55.3)	0 (0)/6 (100)	**0.022**
Height (cm)	155.0 ± 9.0	160.1 ± 7.9	0.199
Weight (kg)	58.2 ± 11.2	63.1 ± 3.7	0.224
BMI (kg/m^2^)	24.1 ± 3.2	24.7 ± 2.3	0.771
Osteoporosis, *n* (%)	7 (18.4)	1 (16.6)	1.000
Diabetes mellitus, *n* (%)	5 (13.1)	1 (16.6)	1.000
Stem alignment (degree)	3.1 ± 3.7	6.4 ± 2.8	**0.033**
FR pro (%)	81.4 ± 7.1	78.0 ± 2.2	**0.040**
FR met (%)	46.1 ± 5.9	42.6 ± 7.1	0.293
FR dia (%)	40.8 ± 6.8	36.9 ± 4.8	0.172

Continuous variables are presented as mean ± standard deviation and compared using the Wilcoxon rank-sum test. Categorical variables are presented as number (percentage) and compared using Fisher’s exact test. BMI, body mass index; FR, filling ratio; pro, proximal; met, metaphyseal; dia, diaphyseal. Bold indicates statistical significance (*p* < 0.05).

**Table 4:** Association between stem alignment and stem subsidence.

	Subsidence		
	No	Yes	Total
SA < 5.5 degrees	31	1	32
SA ≥ 5.5 degrees	7	5	12
Total, *n*	38	6	44

Subsidence is significantly more frequent when stem alignment (SA) was ≥5.5°. (*p* < 0.0037 by Fisher’s exact test).

**Table 5:** Association between proximal filling ratio and stem subsidence.

	Subsidence		
	No	Yes	Total
FR pro < 80%	15	6	21
FR pro ≥ 80%	23	0	23
Total, *n*	38	6	44

The relative risk of stem subsidence is significantly higher when filling ratio (FR) pro < 0.8. (*p* < 0.0077 by Fisher’s exact test).

**Table 6:** Independent predictors of stem subsidence using logistic regression analysis.

Predictor	OR	95% CI	*p* Value
FR pro	70	3.6–1342.6	<0.01
Stem alignment	0.031	0.99–1.20	0.99

Filling ratio (FR) pro is a significant independent predictor of subsidence. In contrast, stem alignment is not. Bold indicates statistical significance (*p* < 0.05).

## Data Availability

The datasets used and/or analyzed during the current study available from the corresponding author on reasonable request.

## Abbreviations

FR	Filling ratio
RSA	Reverse shoulder arthroplasty
USSPs	Uncemented short stem prostheses
ROM	Range of motion
OR	Odds ratio
CI	Confidence interval
ROC	Receiver operating characteristic
CT	Computed tomography
